# Secretome Profiling of Young Multipotent Stem Cells Reveals Angiogenic and Immunomodulatory Mechanisms Supporting Aged Neuromuscular Health

**DOI:** 10.1111/acel.70408

**Published:** 2026-02-12

**Authors:** Seth D. Thompson, Chelsea L. Rugel, Maddlyn R. Haller, Jodi L. Curtin, Sudarshan Dayanidhi, Mitra Lavasani

**Affiliations:** ^1^ Shirley Ryan AbilityLab Chicago Illinois USA; ^2^ Department of Physical Medicine and Rehabilitation Northwestern University Chicago Illinois USA; ^3^ Northwestern University Interdepartmental Neuroscience (NUIN) Graduate Program Northwestern University Chicago Illinois USA

**Keywords:** aging, angiogenesis, immune‐modulation, neuromuscular diseases, rejuvenation, secretome, stem cells, systemic transplantation

## Abstract

Aging is the primary risk factor for many neuromuscular (NM) diseases that impair motor and cognitive function. Transplantation of young muscle‐derived stem/progenitor cells (MDSPCs) has shown remarkable therapeutic potential across a range of age‐related diseases, primarily through paracrine mechanisms. In this study, secretome profiling of young MDSPCs revealed a unique enrichment of pro‐angiogenic and immunomodulatory proteins compared to their aged counterparts. Our systemic transplantation experiments also demonstrate that young MDSPCs activate biological pathways linked to these secreted factors, providing strong mechanistic evidence of their contribution to the reversal of age‐associated NM decline at molecular, structural, and functional levels. Systemic transplantation of young MDSPCs into naturally aged mice enhanced motor function and reduced anxiety‐like behavior. Structural improvements in aged NM tissues were partially mediated by phosphorylating protein sites involved in muscle neovascularization and regulation of blood–brain barrier integrity in the motor cortex. Paracrine signaling from young MDSPCs enhanced the endogenous regenerative capacity of aged tissues, with effects sustained for up to 2 months post‐transplantation. Overall, this study elucidates the molecular basis of MDSPC‐mediated NM rejuvenation and provides a foundation for developing novel protein–based therapies to combat age‐related functional decline.

## Graphical abstract

Young multipotent muscle‐derived stem/progenitor cells (MDSPCs) secrete a unique profile of pro‐angiogenic and immunomodulatory proteins that are diminished with age. Systemic transplantation of young MDSPCs into naturally aged mice activates pathways in neuromuscular tissues, enhancing health and driving molecular, structural, and functional rejuvenation. Diagram was created with BioRender.com.

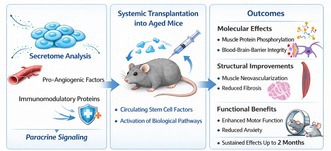

## 1 Introduction

One of the nine hallmarks of aging is altered intercellular communication (Lopez‐Otin et al. 2013). Normally, intercellular signals that promote tissue regeneration are secreted molecules released by resident progenitor cells (Charge and Rudnicki 2004; Chambers and Mcdermott 1996; Brochhausen et al. 2009; Lavasani et al. 2011). However, as tissue homeostasis declines with age, it leads to the dysregulation of key signaling pathways—including those involved in angiogenesis and inflammation—that affect neuromuscular (NM) tissues (Jia et al. 2019; Ernst et al. 1990; Miller 1996). Indeed, aged muscle exhibits a significant reduction in vascularization and an impaired ability to endogenously form new vessels (Cooper et al. 1994). Similarly, breakdown of the blood brain barrier (BBB) in the aged brain, including reduced endothelial cell volume, pericyte and astrocyte connections, and blood turnover, compromises the ability to regulate a healthy vascular environment (Knox et al. 2022). Augmenting angiogenic paracrine signaling, with transgenic models or viral transduction, has been shown to not only stimulate neovascularization but also rejuvenate several NM tissues (Grunewald et al. 2021). In addition, regulation of immune signaling becomes compromised with age, due to the loss of “speed bump” mechanisms that keep the immune system in check (Carpentier et al. 2013; Bruunsgaard et al. 2003). Immune dysregulation is associated with frailty and mortality (Schaap et al. 2009; Pedersen et al. 2003) and can lead to or accelerate a wide array of age‐related diseases (Schaap et al. 2009; Pedersen et al. 2003; Greten and Grivennikov 2019; Kapoor et al. 2011; Lyra et al. 2021; Marogianni et al. 2020; Spagnuolo et al. 2018). In muscle, increased inflammation also stimulates an expression profile in resident fibroblasts that leads to excessive collagen deposition leading to reduced repair and increased fibrosis (Munoz‐Canoves and Serrano 2015). Frequently, tissue degradation initially from an injury, disuse, or other aging‐related chronic disease, will activate inflammation, which in turn increases expression of catabolic factors, leading to further degradation and a destructive positive feedback loop that is difficult to suppress (Carpentier et al. 2013; Bruunsgaard et al. 2003; Loeser Jr. 2004; O'mahony et al. 1998).

While blood product treatments, including platelet rich plasma (PRP) or hyperacute serum, have shown some potential in vitro for increasing stem cell proliferation and ameliorating phenotypes associated with chronic inflammation, their application in vivo has yielded poor results, particularly in older patients whose regenerative capacity is already diminished (Moussa et al. 2017; Kardos et al. 2019; Li et al. 2013; Wang 2014). Similarly, systemic factors from young mice have been shown to increase muscle regeneration in aged mice following shared vasculature in heterochronic parabiosis and similar results were observed when aged muscle was grafted into young hosts, but the underlying mechanisms remain elusive. While these experiments provide valuable mechanistic insights (Conboy et al. 2005; Conboy and Rando 2012; Liu et al. 2017; Brack et al. 2007; Carlson and Faulkner 1989), treatments relying on allogenic patient donations (i.e., blood, serum, and plasma treatments) do not have the scaling potential to meet the growing need of the aging population, making cellular or pharmaceutical therapies more attractive alternatives.

Previously, we discovered that systemic transplantation of young muscle‐derived stem/progenitor cells (MDSPCs) into mouse models of progeria promotes significant healthspan and lifespan extension (Lavasani et al. 2012). Importantly, “old” MDSPCs, isolated from progeria or naturally aged mice, did not increase healthspan or lifespan. We also showed that young MDSPCs functionally rescued intrinsic defects—including proliferation and myogenic differentiation—of old MDSPCs in an in vitro coculture system through secreted factors (Lavasani et al. 2012; Song et al. 2013). More recently, we have demonstrated that systemic transplantation of young MDSPCs into progeroid mice provides a sex‐specific preservation of female NM tissues and muscle endurance (Thompson et al. 2024). In addition, systemic transplantation of young MDSPCs into naturally aged mice—the same cohort analyzed for this study—is able to improve movement and gait, reduce chronic inflammation in articular cartilage, and increase regenerative chondrogenic markers while reversing osteoarthritis‐related pathologies (Thompson, Pichika, Lieber, Budinger, and Lavasani 2021). Notably, donor cells were not found within the rejuvenated neuromusculoskeletal tissues during any of these experiments, indicating that young MDSPCs secrete factors that modulate pathways involved with rejuvenating aged tissues. However, which therapeutic factors young MDSPCs differentially secrete compared to old MDSPCs, and what molecular pathways are targeted, are unknown.

Here, we tested the hypothesis that the proteins differentially secreted by young MDSPCs, compared to old MDSPCs, are enriched with factors that promote rejuvenation of aged NM tissues. We used an antibody array to quantify proteins secreted by young and old MDSPCs and analyzed NM tissues in naturally aged mice systemically transplanted with young MDSPCs to investigate pathways related to these factors. Our results demonstrate that MDSPCs secrete factors that modulate angiogenesis and immune signaling pathways directly related to functional and histopathological improvements we observed in vivo following systemic injection. The identification of these factors will provide a foundation for a more targeted and clinically applicable intervention to improve NM health and function in older adults.

## 2 Results

### 2.1 Young MDSPCs Secrete Pro‐Angiogenic and Immune‐Modulatory Factors

**FIGURE 1:**
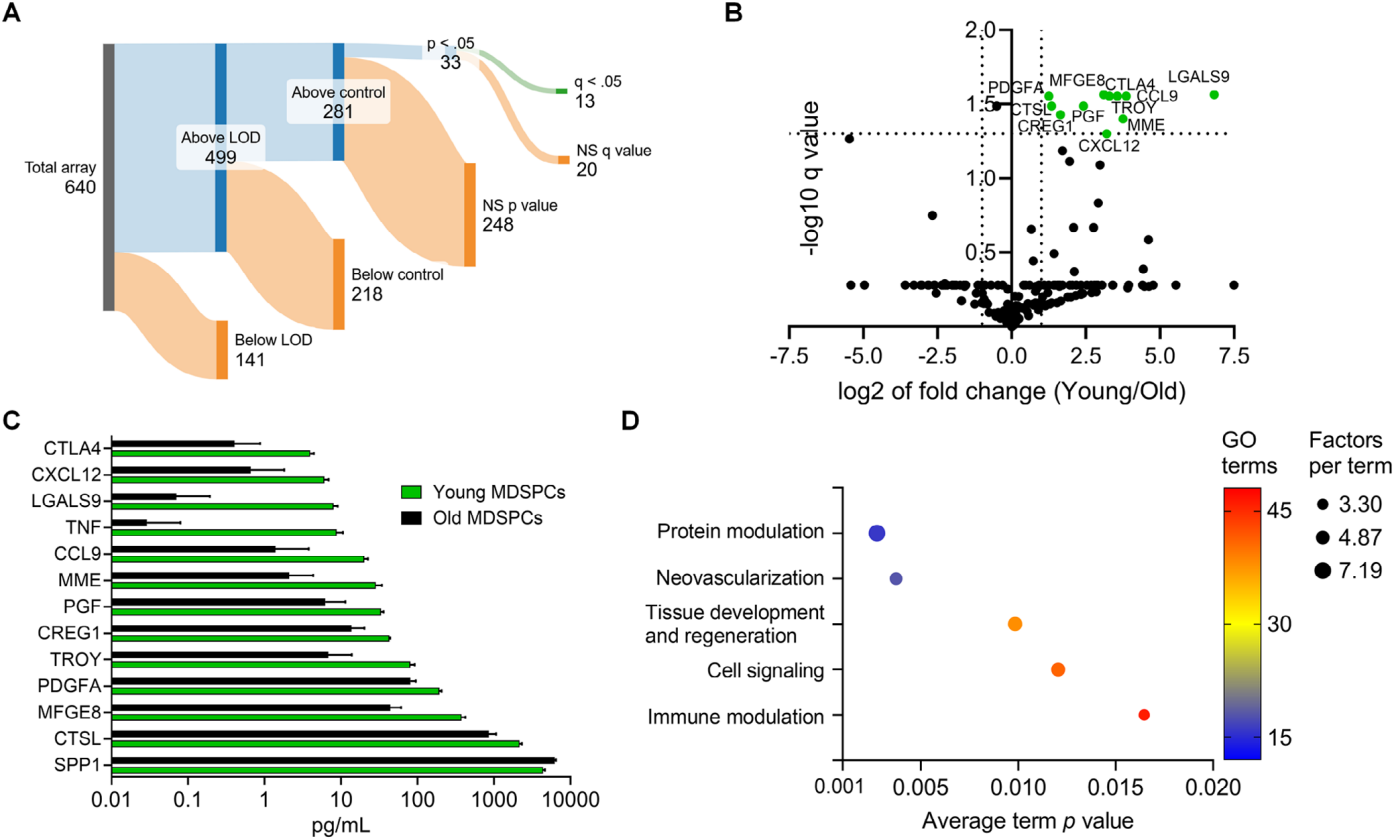
Protein quantification and analysis of young and old MDSPC secretomes. Proteins secreted by young and old MDSPCs were measured using a glass slide antibody array targeting 640 unique proteins. (A) The analytical workflow leading to the identification of 13 significantly different secreted factors (adjusted *p*‐values, *q* ≤ 0.05) is illustrated by a Sankey diagram. (B) Volcano plot showing differentially secreted proteins from young and old MDSPCs. Twelve proteins (green) have a > 2 fold difference (*x*‐axis, dotted lines) and *q* ≤ 0.05 (*y*‐axis dotted line), all of which were upregulated in the young MDSPC secretome. (C) Protein concentrations of each significantly differentially secreted factor by young (green) and old (black) MDSPCs. (D) Dot plot showing the key categories supported by proteins enriched in the young MDSPC secretome, based on significantly enriched gene ontology (GO) terms. Sankey diagram was created with sankeymatic.com.

We first aimed to identify proteins secreted by young MDSPCs and what molecular mechanisms these protein factors are most likely to affect. The same population of young MDSPCs used in our previous rejuvenation studies was chosen for secretome analysis (Lavasani et al. 2012; Thompson, Pichika, Lieber, Budinger, and Lavasani 2021; Thompson, Pichika, Lieber, and Lavasani 2021). Culture media collected from young MDSPCs and populations of MDSPCs isolated from 2‐year‐old naturally aged mice (old MDSPCs) were used as a comparative model. Of the 640 cytokines and growth factors tested, 499 proteins had detectable levels from at least one sample and 281 had quantifiable levels from experimental samples (young or old MDSPCs) above the media alone control (Figure 1A). The mean values of these 281 proteins were used for statistical analysis to compare the secretome of young MDSPCs against old MDSPCs. Using the false discovery rate (Benjamini and Hochberg 1995), 13 factors were defined as significantly different (*q* value ≤ 0.05) between groups.

The *q* values for each factor were plotted against their fold change between groups and graphed as a volcano plot (Figure 1B). Twelve out of the 13 identified factors were elevated over 2‐fold in the secretome of young MDSPCs. The factors secreted at significantly greater quantities (Figure 1C) by young MDSPCs were galectin‐9 (LGALS9), milk fat globule‐EGF factor 8 (MFGE8, aka lactadherin), cytotoxic T‐lymphocyte‐associated protein 4 (CTLA4), platelet‐derived growth factor subunit A (PDGFA), chemokine ligand 9 (CCL9, aka MIP1γ), tumor necrosis factor receptor superfamily member 19 (TNFRSF19, aka TROY), cathepsin L (CTSL), placental growth factor (PGF), tumor necrosis factor (TNF), cellular response of E1A‐stimulated genes 1 (CREG1), membrane metalloendopeptidase (MME, aka neprilysin), and stromal cell‐derived factor 1 (CXCL12, aka SDF1). The only factor significantly elevated in old MDSPCs was osteopontin (SPP1, aka OPN), which was not expressed above a 2‐fold increase. The 12 factors secreted at higher quantities by young MDSPCs were used for functional annotation and enrichment analysis using the Database for Annotation, Visualization, and Integrated Discovery (DAVID) online bioinformatics resource with the 281 analyzed proteins as a reference. Over 250 gene ontology (GO) terms were significantly enriched, with the majority falling under broader categories such as protein modulation, neovascularization, tissue development and regeneration, cell signaling, and immune modulation (Figure 1D). The secretome profiling of young MDSPCs reveals a proteomic signature enriched in angiogenic and immune‐modulatory factors.

### 2.2 Systemic Transplantation of Young MDSPCs Improves Motor Function in Naturally Aged Mice

**FIGURE 2:**
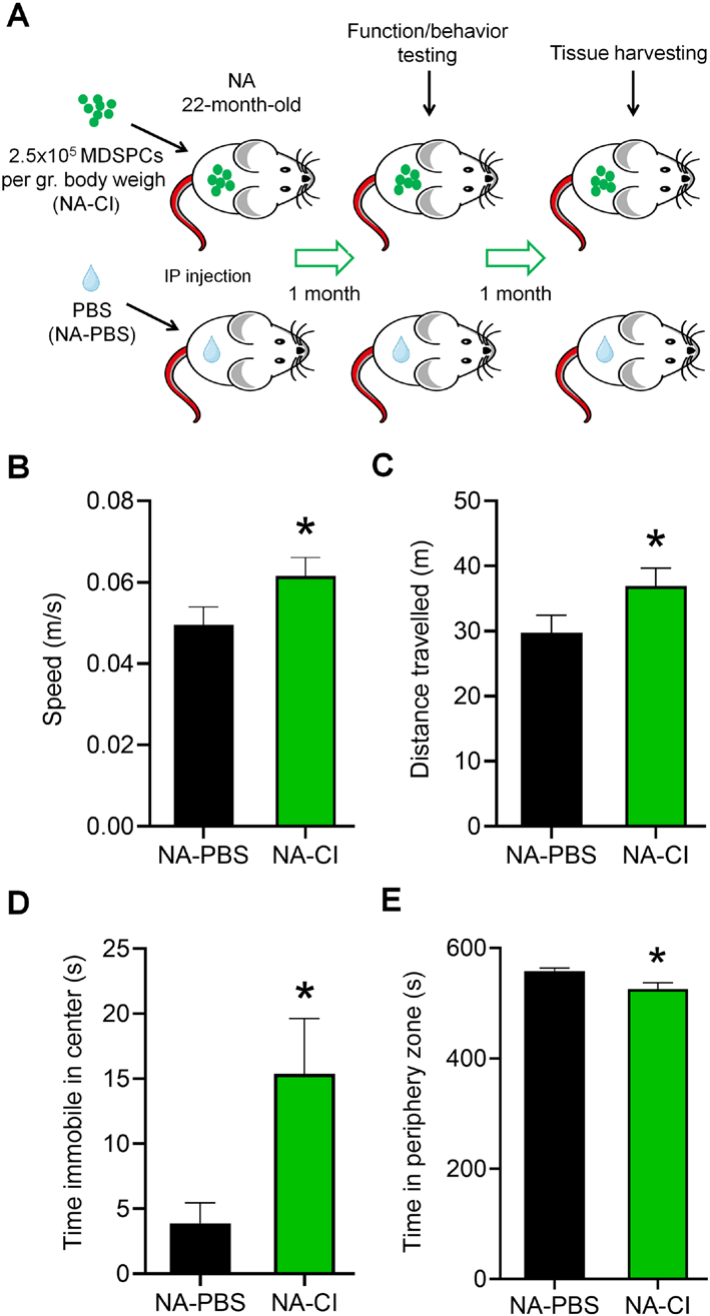
Functional and behavioral improvements in cell treated mice assessed by the open field test. (A) Diagram showing the in vivo experimental design. Naturally aged mice were intraperitoneally injected with either young MDSPCs (NA‐CI) or vehicle PBS (NA‐PBS) at 22 months of age. One month following treatment, mice were evaluated using the open field test to assess locomotor activity and anxiety‐like behavior. Graphs show (B) average speed and (C) total distance traveled by mice systemically injected with young MDSPCs (NA‐CI; *n* = 10) or PBS (NA‐PBS; *n* = 8). (D) Time spent immobile in the center zone and (E) total time spent in the periphery zone during the 10‐min test are shown. Data are presented as mean ± SEM (B). *p ≤ 0.05 using one‐tailed unpaired Student's *t*‐test. Diagram was created with BioRender.com.

Naturally aged mice were intraperitoneally injected at 22 months of age with either young MDSPCs (cell injected, NA‐CI) or PBS as vehicle (NA‐PBS). Mice were functionally assessed 1 month post‐transplantation and euthanized 2 months post‐transplantation for tissue collection (Figure 2A). Functional and behavioral changes were evaluated 1 month after young MDSPC transplantation using a 10‐min open field test. During the test, NA‐CI mice had significantly more spontaneous movement, traveling at an average speed (Figure 2B) of 0.0615 m/s (±0.0046) compared to 0.0495 m/s (±0.0045) by NA‐PBS mice, and therefore traveling an average distance of 36.91 m ± 2.784 by NA‐CI mice compared to 29.75 m ± 2.694 by NA‐PBS mice (Figure 2C). This data supports our previously published results from the same cohort of mice, which demonstrated that NA‐CI mice were significantly more capable of running at higher speed on a Digigait treadmill and exhibited notable improvement in gait metrics associated with age‐related movement disorders (Thompson, Pichika, Lieber, Budinger, and Lavasani 2021). Interestingly, behavioral aspects of the open field test, including time spent immobile in the center (Figure 2D) and time spent in the periphery zone (Figure 2E), were also significantly different between NA‐CI and NA‐PBS cohorts. NA‐CI mice spent significantly more time in and immobile in the center zone (74.03 s ± 11.2 and 15.36 s ± 4.3, respectively) compared to NA‐PBS mice (41.79 s ± 5.3 and 3.875 s ± 1.6, respectively). In contrast, NA‐PBS mice spent more time along the periphery of the testing area (558.21 s ± 5.3) compared to NA‐CI mice (525.97 s ± 11.2), suggesting reduced levels of anxiety in MDSPC transplanted mice.

### 2.3 Systemic Transplantation of Young MDSPCs Enhances Skeletal Muscle Neovascularization and Modulates Pathways of Vessel Integrity

**FIGURE 3:**
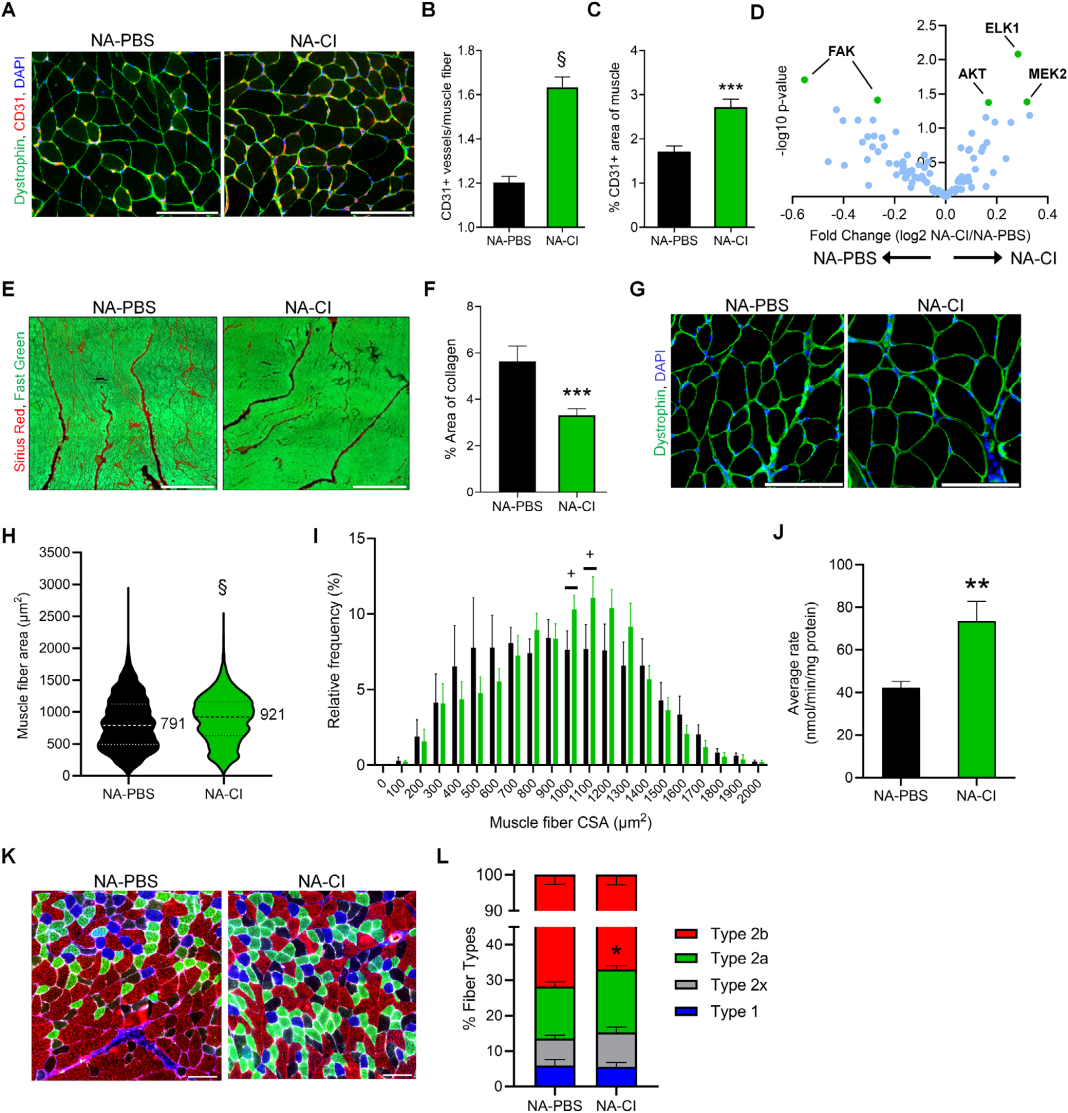
Systemic transplantation of young MDSPCs promotes skeletal muscle neovascularization, improves muscle structure, and increases mitochondrial content. (A) Representative immunohistochemical images of gastrocnemius (GS) muscles from young MDSPC (NA‐CI; *n* = 9) and PBS (NA‐PBS; *n* = 7) treated mice, labeled for CD31 (red) and dystrophin (green). (B) Quantification of vasculature density, presented as the number of vessels per muscle fiber. (C) Quantification of total vasculature area, presented as percentage of muscle area occupied by vessels. (D) Volcano plot depicting statistical significance and fold change of neovascularization pathway protein phosphorylation levels in GS muscles from NA‐CI (*n* = 4) and NA‐PBS (*n* = 4) mice. Proteins that have sites with significantly increased phosphorylation in NA‐CI muscles are highlighted in green. (E) Representative images of GS muscles stained with Sirius red (collagen, red) and Fast Green (muscle, green) at 2 months post‐intraperitoneal (IP) transplantation. (F) Quantification of collagen content as a percentage of total tissue area. (G) Representative images of dystrophin‐labeled GS muscles (green) from NA‐CI and NA‐PBS mice. (H) Violin plot of muscle fiber cross‐sectional area (CSA), with lines indicating median and interquartile ranges (25th and 75th percentiles). (I) Frequency distribution of muscle fiber CSA binned in 100 μm^2^ intervals. (J) Citrate synthase activity measured in quadriceps muscles of NA‐CI (*n* = 9) and NA‐PBS (*n* = 7) mice. (K) Representative images of GS muscles labeled for type I (blue), type IIa (green), and type IIb (red) muscle fibers at 2 months post‐IP transplantation. (L) Stacked bar plot of fiber type composition in GS muscles of NA‐CI (*n* = 7) and NA‐PBS (*n* = 7) mice. Data (B, C, F, I, J, L) are presented as mean ± SEM. **p ≤ 0.01, ***p ≤ 0.001, and ^§^ *p* ≤ 0.0001 using one‐tailed unpaired Student's *t*‐test. (H) ^§^ *p* ≤ 0.0001 by two‐tailed Kolmogorov–Smirnov test. Scale bars are 100 μm (A, G, K) and 500 μm (E).

Mice were euthanized 2 months post‐transplantation at 24 months of age and the mean terminal body weights of the NA‐CI (27.74 g ± 1.04) and NA‐PBS (29.17 g ± 1.54) cohorts were not significantly different (Figure S1A). Based on pathway analysis from our young MDSPC secretome data, we investigated the potential effects of factors supporting neovascularization in muscle and brain tissues of NA‐CI mice following systemic transplantation. Gastrocnemius (GS) muscle sections were labeled for dystrophin (green) to highlight muscle fiber perimeters and CD31 (red) to identify endothelial cells within microvessels (Figure 3A). Our results indicate that the GS muscles from NA‐CI mice contained a significantly greater number of CD31+ vessels (1.63 ± 0.05, *n* = 9) per muscle fiber than NA‐PBS (1.20 ± 0.03, *n* = 8), displaying a greater density of muscle vasculature (Figure 3B). The % area of each muscle consisting of vasculature was also significantly greater in NA‐CI muscles (2.72% ± 0.18%) compared to NA‐PBS muscles (1.72% ± 0.13%; Figure 3C).

To gain a deeper mechanistic understanding of the pathway activation that increased the muscle vasculature observed in transplanted mice, a protein phosphorylation antibody array targeting proteins purported to be involved in the neovascularization pathway was performed on GS muscle tissue of NA‐CI (*n* = 4) and NA‐PBS (*n* = 4) mice. Five phosphorylation sites were found to be significantly different between groups (Figure 3D). Phosphorylation of ELK1 (Ser389), MEK2 (Thr394), and AKT (Thr308)—all of which have established roles in the neovascularization pathway (Guo et al. 2021; Liang et al. 2021; Kevil et al. 2004; Dossumbekova et al. 2008)—was significantly higher in NA‐CI muscles compared to NA‐PBS. In contrast, phosphorylation of FAK at Tyr576 and Ser910—sites with conflicting or less well‐defined roles in the pathway (Rasmussen et al. 2015; Shikata et al. 2003)—was significantly elevated in NA‐PBS muscles compared to NA‐CI. Greater levels of protein phosphorylation on sites known to support neovascularization provide mechanistic insight into how young MDSPCs convey therapeutic benefits.

### 2.4 Systemic Transplantation of Young MDSPCs Improves Aged Muscle Structure and Mitochondrial Function

Levels of fibrosis in skeletal muscle have been shown to increase with inflammation caused by injury or disease (Munoz‐Canoves and Serrano 2015). Therefore, muscle fibrosis—as measured by % area of collagen (red) from muscle (green) cross sections—was analyzed to assess structural extracellular matrix (ECM) changes in treated and control aged mice (Figure 3E). GS muscle sections of NA‐PBS mice were found to contain significantly greater levels of collagen (5.6% ± 0.67%, *n* = 7) compared to NA‐CI mice (3.3% ± 0.28%, *n* = 9; Figure 3F). In addition, collagen fibrils are denser than muscle fibers (Gillies and Lieber 2011), which means a greater proportion of the NA‐PBS GS muscle wet weights is attributed to collagen than in NA‐CI muscles.

To investigate potential mechanisms related to the functional changes, the cross‐sectional area (CSA) of muscle fibers (green), which has been shown to directly correlate with muscle force generation (Wood et al. 1975), was measured from GS muscles (Figure 3G). Muscle fiber CSA distributions from NA‐CI and NA‐PBS GS muscles were determined to be statistically different using a two‐tailed Kolmogorov–Smirnov test. NA‐CI muscle fibers were larger with a median and 25th–75th percentiles of 921 and 629–1161 μm^2^, compared to NA‐PBS muscle fiber CSA with a median and 25th–75th percentiles of 791 and 492–1126 μm^2^ (Figure 3H). To further illustrate the distribution of dystrophin‐positive muscle fiber CSA, data were grouped into 100 μm^2^ bins and presented as cohort averaged frequency distributions (Figure 3I). Quantitative comparisons revealed that NA‐CI muscles exhibit a trend toward a higher percentage of larger fibers in the 1000 and 1100 μm^2^ bins compared to NA‐PBS muscles (^+^ *p* ≤ 0.07). However, GS muscle wet weights (Figure S1B), normalized to animal body weights, were not statistically different in NA‐CI mice (0.888% ± 0.10%) compared to NA‐PBS mice (0.843% ± 0.08%).

Since mitochondrial function decreases with age (Gonzalez‐Freire et al. 2018), we investigated whether muscle function improvements following MDSPC therapy are related to increased mitochondrial function. Therefore, mitochondrial citrate synthase rates—a faithful surrogate for evaluating mitochondrial content (Larsen et al. 2012)—and mitochondrial respiration were measured in quadriceps muscles of treated and control mice. Quadriceps muscles from NA‐CI mice demonstrated significantly higher citrate synthase rates compared to NA‐PBS muscles (73.5 ± 8.9, *n* = 9 and 42.2 ± 3.1 nmol/min/mg protein, *n* = 7, respectively), indicating higher energy production (Figure 3J). While rates of mitochondrial respiration were greater in NA‐CI muscles at each respirometry state, compared with NA‐PBS muscles, these differences were not statistically significant (Figure S1C).

Muscle mitochondrial analyses revealed greater changes in mitochondrial content than respiration capacity. Therefore, we performed muscle fiber‐type analysis to determine whether fiber‐type shifts could shed light on this observation (Figure 3K). Indeed, the percentage of type IIa muscle fibers was significantly increased in GS muscles of NA‐CI mice compared with NA‐PBS controls (17.9% ± 1.0, *n* = 7 and 14.7% ± 1.3%, respectively, *n* = 7 per group; Figure 3L), which are fatigue‐resistant fibers containing greater quantities of mitochondria. This increase in type IIa fibers appears to occur primarily at the expense of fast fatigable type IIb fibers in NA‐CI muscles compared with NA‐PBS muscles (66.9% ± 2.8% vs. 71.8% ± 2.7%, respectively), which have lower mitochondria abundance. Together, these results suggest that young MDPSC treatment confers improvements to muscle function, in part, by improving muscle histopathology and mitochondrial content.

To better understand the mechanism of action, we asked whether the functional improvement resulted directly from young MDSPC cellular engraftment and activity versus their circulating secreted factors. Since young MDSPCs were transduced with a retroviral vector containing *LacZ* with a nuclear localization sequence (*nLacZ*), we analyzed 10 tissues for the presence of donor cells. Importantly, similar to our previous findings in progeria and naturally aged mice (Thompson et al. 2024; Lavasani et al. 2012), donor cells were not detected in any peripheral tissues of recipient mice 2 months post‐transplantation (Figure S2), supporting that the therapeutic effects of MDSPCs are mediated by systemically acting secreted factors.

### 2.5 Systemic Transplantation of Young MDSPCs Increases Neovascularization and Enhances Blood–Brain Barrier Integrity of Aged Motor Cortex

**FIGURE 4:**
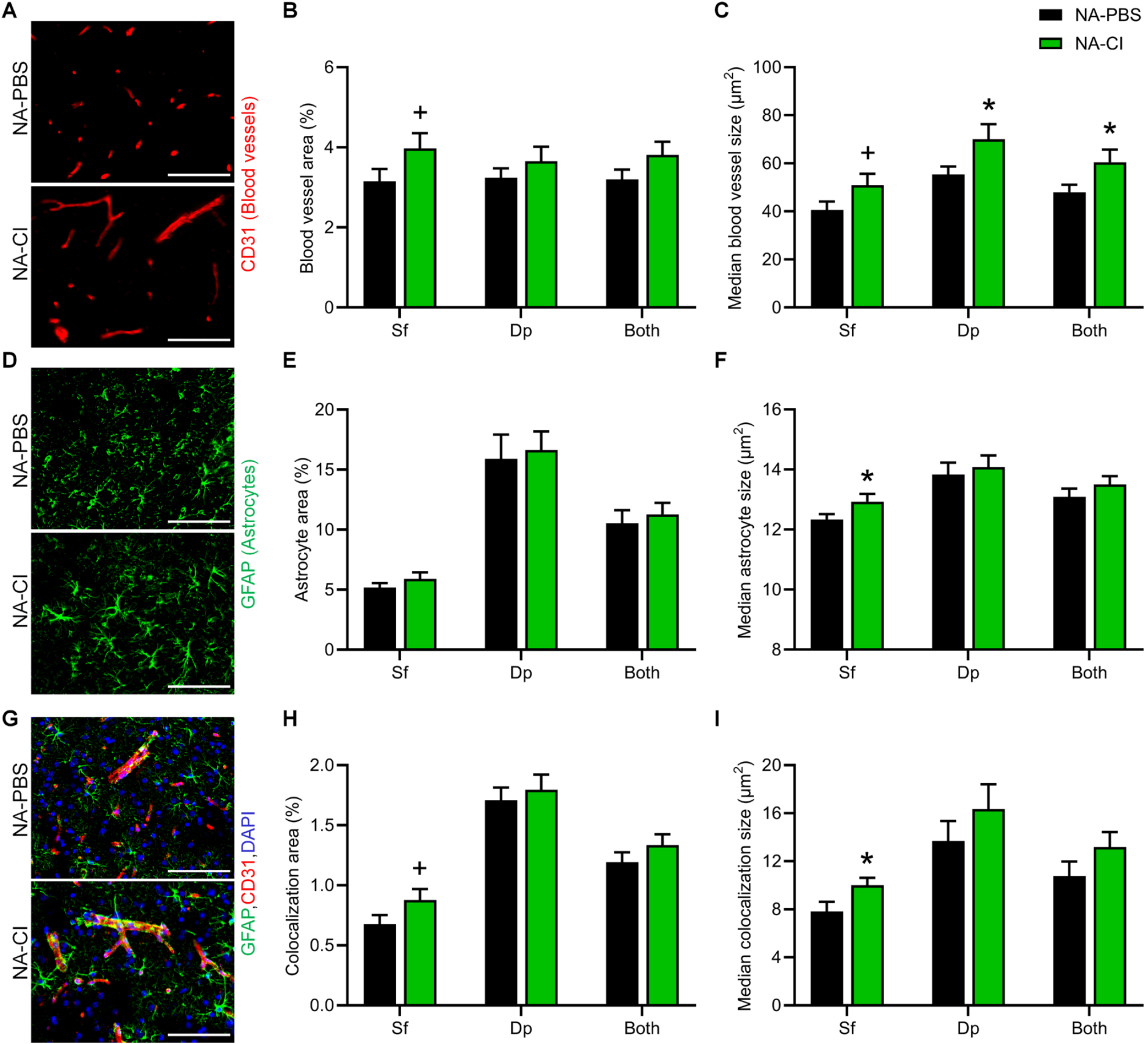
Systemic transplantation of young MDSPCs induces motor cortex neovascularization and improves blood–brain barrier integrity. (A) Representative immunohistochemical images of deep (Dp) motor cortical layers (6a and 6b) from young MDSPC (NA‐CI; *n* = 9) and PBS (NA‐PBS; *n* = 7) injected mice labeled for CD31+ blood vessels (red). (B) Quantification of blood vessel area as percentage of total region of interest (ROI). (C) Quantification of median blood vessel size. (D) Representative images of superficial (Sf) motor cortical layers (layers 2/3 and 5) labeled for GFAP+ astrocytes (green). (E) Quantification of GFAP+ astrocyte area as a percentage of total ROI. (F) Quantification of median astrocyte size. (G) Representative images of superficial motor cortical layers labeled for both CD31 (red) and GFAP (green). (H) Quantification of percent CD31 and GFAP signal colocalization area relative to ROI. (I) Quantification of median CD31 and GFAP signal colocalization size. Data are presented as mean ± SEM. *p ≤ 0.05, + indicates a trend (*p* ≤ 0.07), using one‐tailed unpaired Student's *t*‐test. Scale bars are 100 μm.

To determine whether systemic implantation of young MDSPCs increased neovascularization in the motor cortex and improved BBB integrity, we quantified CD31+ blood vessels (Figure 4A) and GFAP+ astrocytes. Given that cortical astrocyte density and morphology vary by proximity to white matter tracts (Bayraktar et al. 2020; Lanjakornsiripan et al. 2018), the motor cortex was subdivided into two distinct regions of interest (ROIs)—superficial (Sf; cortical layers 2/3 and 5) and deep (Dp; cortical layers 6a and 6b).

The superficial motor cortex showed a trend toward increased percent area of CD31+ blood vessels in NA‐CI mice (3.97% ± 1.15%, *n* = 9) compared to NA‐PBS mice (3.16% ± 0.81%, *n* = 7, *p* ≤ 0.07; Figure 4B). Across all cortical regions (superficial, deep, and combined), the median size of individual blood vessels was significantly larger in NA‐CI (60.50 ± 5.23 μm^2^) than in NA‐PBS mice (48.00 ± 3.12 μm^2^, Figure 4C), indicating enhanced vascularization. While overall percent area of GFAP+ astrocytes (Figure 4D) did not differ between groups (Figure 4E), the median size of astrocytes in the superficial cortex of NA‐CI mice was significantly larger (5.90 ± 0.54 μm^2^) compared to NA‐PBS mice (5.19 ± 0.36 μm^2^; Figure 4F). Furthermore, percent colocalization area of GFAP+ astrocytes with CD31+ blood vessels (Figure 4G)—an indicator of neurovascular unit integrity—trended higher in the superficial cortex of NA‐CI mice (0.88% ± 0.09%) versus NA‐PBS mice (0.68% ± 0.08%; Figure 4H), and had significantly larger median colocalization size (NA‐CI: 10.00 ± 0.62 μm^2^, NA‐PBS: 7.82 ± 0.80 μm^2^; Figure 4I). These findings suggest that systemic delivery of young MDSPCs enhances cortical neovascularization and may contribute to BBB restoration—particularly in superficial cortical layers—through secretion of paracrine pro‐angiogenic factors.

## 3 Discussion

This is the first comprehensive study that uncovers the factors secreted by young multipotent adult MDSPCs and identifies mechanisms underlying their remarkable ability to systemically rejuvenate NM tissues. Given that the therapeutic effects of MDSPCs have been shown to be mostly mediated by the paracrine factors they secrete (Song et al. 2013; Lavasani et al. 2012; Thompson et al. 2024; Thompson, Pichika, Lieber, Budinger, and Lavasani 2021; Thompson, Pichika, Lieber, and Lavasani 2021), we aimed to elucidate the composition of the young MDSPC secretome using a multiplex antibody array. Here we discovered the secreted proteome unique to young MDSPCs compared to their aged counterparts. Computational pathway analysis predicted that the secreted proteins regulate many processes involved in vascularization and immune modulation. The factors especially relevant for significant pro‐angiogenesis GO terms such as “vasculature development” are PGF, CXCL12, MFGE8, PDGFA, and TNF. Notably, PGF, a member of the VEGF subfamily, plays a critical role in vasculogenesis and angiogenesis, particularly in regenerative contexts (Carmeliet et al. 2001). In addition, MFGE8 has been shown to promote endothelial cell survival and vascular remodeling (Silvestre et al. 2005), while CXCL12 is a key chemokine involved in the recruitment of endothelial progenitor cells (Zheng et al. 2007). Together, these factors may contribute to the increased vascularity observed in distal tissues following intraperitoneal delivery of muscle stem cells. The factors most linked to immune‐modulatory GO terms such as “regulation of leukocyte differentiation” were CTLA4, CCL9, LGALS9, and TNF. CTLA4 is a circulating immune regulator that attaches to the CD86 receptor of antigen presenting cells (APCs), which normally bind and activate T‐cells through their CD28 receptors (June et al. 1989), which triggers T‐cell proliferation and production of pro‐inflammatory IL‐2. Interrupting this APC to T‐cell binding is particularly relevant for reducing age‐related inflammation because T‐cell responsiveness to IL‐2 increases with age (Pekalski et al. 2013). The timing‐dependent effects of LGALS9, CTLA‐4, and other secreted factors are important to consider. While the anti‐fibrotic and anti‐inflammatory actions of these proteins are expected to occur primarily at early time points (1–2 weeks) (Arikawa et al. 2010), early reductions of inflammatory signaling can initiate downstream cascades that support sustained improvements in neuromusculoskeletal tissues. CCL9 primarily functions as a chemokine involved in immune cell recruitment and is therefore often associated with pro‐inflammatory contexts without acting as a direct inflammatory trigger. It has also been implicated in osteoclast signaling (Lean et al. 2002) and identified as a marker of progenitor cells supporting bone regeneration (Nakayama et al. 2022). However, CCL9 remains poorly characterized in the context of aging, making its role in the MDSPC secretome difficult to define.

Since young MDSPCs were previously shown to reduce chronic inflammation in naturally aged mice (Thompson, Pichika, Lieber, Budinger, and Lavasani 2021), it's particularly surprising to observe the involvement of TNF—an immune‐modulatory factor commonly associated with increased inflammation and apoptotic cell death. However, under certain conditions, TNF is also known to mediate cell survival and immune tolerance (Gupta et al. 2003; Sun 2012). In this context, results highlight TNF signaling through the non‐canonical NF‐κB signaling pathway specifically via RelB/p52, rather than the canonical pro‐inflammatory p65/p50 pathway (Sun 2012). TNF can signal through both the canonical and non‐canonical NF‐κB pathways, with the specific route depending on the presence of various activators or inhibitors, and consequently, which receptor is engaged—TNFR1 or TNFR2. Non‐canonical signaling through TNFR2 is known to promote immune tolerance to self‐antigens, downregulate pro‐inflammatory cytokines, support cell survival, and maintain bone homeostasis (Sun 2012; Tas et al. 2007). Indeed, gene ontology analysis lists TNF's involvement in terms enriched by the young MDSPC secretome such as “Regeneration,” “Vascular Development,” “Angiogenesis,” “Blood Vessel Morphogenesis,” and “Animal Organ Development.” Therefore, although TNF is widely recognized as a pro‐inflammatory cytokine and would not be considered a therapeutic candidate in the context of aging, its functions are highly context dependent. Thus, it is possible that young MDSPCs exert systemic effects via the TNFR2‐mediated non‐canonical pathway, promoting immune tolerance and cell survival—two key functions frequently disrupted with age. The identified secreted factors that promote angiogenesis and immune modulation are supported as rejuvenation effectors by our current data, in line with our previously published findings showing increased angiogenesis in a mouse model of progeria (Lavasani et al. 2012) and reduced inflammation in articular cartilage of naturally aged and progeroid mice (Thompson, Pichika, Lieber, Budinger, and Lavasani 2021; Thompson, Pichika, Lieber, and Lavasani 2021). These angiogenesis and immune modulation signaling pathways, which are significantly impaired during aging (Grunewald et al. 2021), appear to be key mechanisms underlying the neuromusculoskeletal rejuvenation observed after systemic transplantation of young MDSPCs, despite the fact that no donor cells could be detected in these tissues.

While no factors were significantly enriched in the old MDSPC secretome beyond a two‐fold increase when compared with young, osteopontin (OPN) was statistically higher even at its 1.4‐fold increase. OPN has been shown to be a pro‐inflammatory senescent‐associates secretory phenotype (SASP) factor, a driver of fibrosis and myocardial aging, and reduced levels of OPN correlate with healthy aging in humans (Sanchis‐Gomar et al. 2016; Sawaki et al. 2018; Shirakawa et al. 2016). These results provide the first insight into the therapeutic molecules secreted by young MDSPCs and reinforce their capacity for effective paracrine signaling.

Structurally, muscles from the cohort treated with young MDSPCs exhibited a significant increase in vasculature. A robust network of blood vessels is essential for supplying oxygen and nutrients to the metabolically active muscle fibers. This functional importance is highlighted by the tendency of muscle nuclei to preferentially locate near vessels during regeneration and by the correlation between capillary density and muscle fiber size being stronger than that with other factors such as oxidative capacity or fiber type (Ralston et al. 2006; Ahmed et al. 1997; Wust et al. 2009; Plyley et al. 1998). Similarly, mitochondria proximity to vasculature enhances mitochondrial capacity and function (Parry et al. 2024). This vasculature‐dependent mechanism likely contributes, at least in part, to the higher citrate synthase activity observed in the quadriceps muscles of young MDSPC‐treated mice. While citrate synthase activity reflects increased mitochondrial content, our respiration measurements suggest that mitochondrial efficiency may also be modestly improved, though not significantly. Together, these findings indicate the enhanced vascularization may underlie both structural and functional improvements during rejuvenation of aged skeletal muscle.

In conjunction with our histological results, further investigation into angiogenic mechanisms revealed significantly elevated phosphorylation at two key angiogenesis‐related protein sites (ELK1[Ser389] and AKT[Thr308]) in muscles of MDSPC‐transplanted mice. These phosphorylation events promote vascular smooth muscle cell proliferation, enhance the production of the vascular stimulating factor eNOS, and contribute to improvements to vascular barrier homeostasis (Guo et al. 2021; Liang et al. 2021; Kevil et al. 2004; Dossumbekova et al. 2008). This may indicate a robust activation of the neovascularization pathway—supported by the histological data—up to 2 months after transplantation. Akt activation also contributes to muscle regeneration (Rommel et al. 2001), reflecting its dual role in promoting angiogenic signaling as well as supporting muscle cell growth and repair. In contrast, the two protein sites with increased phosphorylation in muscles of aged control mice are associated with atherosclerosis and endothelial cell motility (FAK[Tyr576]), or proliferation of epithelial and fibroblast cells (FAK[Ser910]), the latter of which likely relates to the observed increased fibrosis in aged control muscles (Hunger‐Glaser et al. 2003; Jiang et al. 2007; Shikata et al. 2003). The absence of detectable donor cells at later time points supports a mechanism independent of sustained engraftment, consistent with paracrine or systemic effects of MDSPCs. Therefore, we propose that young MDSPCs may promote functional rejuvenation of aged skeletal muscle through secretion of pro‐angiogenic factors, which activate known neovascularization pathways and lead to increased capillary density, thereby better supporting muscle growth and health. Furthermore, the greater levels of protein phosphorylation on sites known to support neovascularization provide mechanistic insight into how young MDSPCs convey therapeutic benefits.

It is interesting to note that while capillary density and muscle fiber size are very closely related (Ahmed et al. 1997; Wust et al. 2009; Plyley et al. 1998), and increased muscle vasculature was observed in cell‐treated mice, this effect did not translate to a comparably robust increase in muscle fiber cross‐sectional areas. A Kolmogorov‐Smirnoff test indicated that the overall distribution of muscle fiber areas differed significantly between MDSPC‐ and PBS‐treated mice; however, specific muscle fiber area ranges in the frequency plot did not reach statistical significance via a Student's *t*‐test. One possible explanation is that fiber‐type shifts and neovascularization occur earlier following transplantation in aged muscle, whereas muscle hypertrophy may develop over subsequent weeks as blood flow and nutrient delivery increase. Additionally, although enhanced vasculature improves oxygenation and the availability of metabolically essential substrates such as glucose, it may not proportionally increase all factors required to drive muscle anabolic activity. Similar considerations may explain the lack of a statistically significant difference in muscle wet weights at this time point. In aged muscles, wet weight reflects not only lean muscle mass but also contributions from components including connective tissue, fibrosis, edema, and inflammatory infiltration. Consistent with this, PBS‐treated aged muscles exhibited increased extracellular matrix content, including fibrosis, which may mask differences in lean muscle mass when assessed by gross weight alone. Although, we have previously reported reduction of muscle fibrosis following systemic transplantation of young MDSPCs in progeroid mice (Lavasani et al. 2012; Ota et al. 2011; Deasy et al. 2005), this is the first report demonstrating reduced fibrosis in naturally aged mice following this treatment. Reduced fibrosis may connect mechanistically with both the observed neovascularization described herein and the reduction of chronic inflammation we previously reported from this same cohort of mice (Thompson, Pichika, Lieber, Budinger, and Lavasani 2021). As previously shown, vascular endothelial cells produce angiotensin 1–7, which attenuates TGF‐β1 signaling, therefore, an increase in vascular endothelium may further reduce TGF‐β1 signaling and decrease skeletal muscle fibrosis (Acuna et al. 2014; Morales et al. 2014). Additionally, chronic inflammation in resident muscle macrophages elevates TGF‐β1 signaling, inhibiting fibro/adipogenic progenitor (FAP) cell apoptosis and promoting collagen deposition (Lemos et al. 2015). When chronic inflammation is reduced, resident macrophages switch to TNF signaling, inducing FAP apoptosis and limiting fibrosis. Thus, systemic reductions in chronic inflammation trigger signal cascades in peripheral tissues, like skeletal muscle, enabling proper anabolic and catabolic regulation (i.e., reduced fibrosis). Similar effects were observed in the articular cartilage of this same cohort of treated mice, as previously reported (Thompson, Pichika, Lieber, Budinger, and Lavasani 2021), where reduced levels of catabolic enzymes and pro‐inflammatory cytokines promoted healthier tissue reconstruction.

The MDSPC‐treated naturally aged mice exhibited greater voluntary mobility and behavior associated with reduced levels of anxiety inside the open field test. Functional and behavioral improvements observed in the open field test—and Digigait analysis previously reported in the same cohort (Thompson et al. 2024)—may also be linked to enhanced angiogenesis and vascular integrity in the motor cortex following systemic transplantation of young MDSPCs. Neurovascular hallmarks of aging, such as reduced blood flow and decreased endothelial cell volume, have previously been ameliorated through heterochronic parabiosis (Katsimpardi et al. 2014). Although MDSPCs do not cross the BBB, they have been shown to stimulate increased vasculature in the brain (Lavasani et al. 2012), suggesting a potential mechanism by which they may influence central nervous system function. Similarly, in this study, naturally aged mice systemically transplanted with young MDSPCs exhibited an increased individual blood vessel size. The improvement of BBB integrity within superficial cortical layers suggests that enhanced vascular support for corticospinal projection neurons—whose cell bodies reside within layer 5 (Munoz‐Castaneda et al. 2021)—may be critical for improving motor function in aged mice. Notable differences were also observed in components of the BBB between treated and control mice. The median colocalization size of astrocytes with blood vessels—where BBB junctions are formed—was significantly higher in the superficial region of the motor cortex in cell treated compared to control mice. Additionally, colocalization percentage area trended higher in the superficial region of cell treated mice. The observed maintenance of BBB integrity and promotion of angiogenesis suggests an improvement in the regulation of nutrient exchange and waste clearance between the motor cortex parenchyma and the circulatory system. Tissue regeneration in aging is a dynamic and temporally heterogeneous process, particularly when compared with acute injury models. Accordingly, future studies will incorporate additional time points and expanded molecular and cellular endpoints—including AQP4 channels, pericytes, and tight junction proteins in the BBB—to better define how systemic MDSPC treatment promotes a healthier microenvironment across NM tissues in naturally aged mice.

Together, our findings demonstrate the rejuvenating effects of systemically transplanted young MDSPCs on aging muscle and brain at the molecular, histological, and functional levels.

Although the multiplex antibody array which quantifies over 600 well‐known circulating proteins provides substantial insights to the secretome of young MDSPCs, it may not capture all rejuvenating factors, including non‐protein mediators or proteins not represented on the array. In addition, we elected not to further process samples to render proteins from vesicles or other complexes—which can unfaithfully alter protein quantification—to maintain sample fidelity of directly bioavailable proteins secreted by MDSPCs but may not detect proteins that are otherwise sequestered (Mukhopadhyay et al. 2016; Bai and Laiho 2012). Moreover, the rejuvenation effect is likely mediated by a combination of identified and unidentified factors, some of which may even be secreted by old MDSPCs, limiting the ability of differential analyses to pinpoint all mediators. Importantly, our in vitro analyses identified multiple candidate secreted factors; however, in vivo evaluation of individual or combinatorial factors will require systematic optimization of dosing and delivery strategies and is therefore planned for future studies.

In this study, PBS‐injected aged animals were used as a well‐established baseline control to assess transplantation‐induced rejuvenation, rather than alternative controls such as injecting old MDSPCs. Extensive prior work using heterochronic parabiosis, blood exchange (Conboy et al. 2005; Rebo et al. 2016; Gonzalez‐Armenta et al. 2021), and transplantation paradigms (Wang et al. 2020; Shen et al. 2011; Lavasani et al. 2012) has demonstrated that exposure to aged systemic environments or aged cells induces deleterious, pro‐geronic effects, including increased cellular senescence, mitochondrial dysfunction, vascular aging, and impaired tissue regeneration. Moreover, dead cells do not mimic an appropriate biological baseline, as they can release pro‐ or anti‐inflammatory mediators (Phulphagar et al. 2021; Rothlin et al. 2021; Korns et al. 2011), depending on the method of cell death, thereby introducing potential normalization bias. Although MDSPC–treated mice exhibited increased activity, we interpret this as a functional outcome of improved NM health rather than a confounding variable, as cohort sizes were sufficient to minimize the likelihood that spontaneous differences in activity account for coordinated improvements observed across muscle, brain, vasculature, and joint tissues (Thompson, Pichika, Lieber, Budinger, and Lavasani 2021).

In summary, our findings indicate that young MDSPCs can modulate the systemic environment of naturally aged animals through secreted rejuvenating factors critical for tissue regeneration. Future studies will verify the direct functional role of the identified proteins in neuromusculoskeletal tissue rejuvenation. A likely translational application of these findings is to test the identified pro‐rejuvenating factors—or existing pharmaceuticals that modulate their molecular pathways—as a combinatorial therapy. Some of the factors are already clinically approved, such as CTLA4 for rheumatoid arthritis (Bluestone et al. 2006), but have not yet been tested as anti‐aging agents. Building on these discoveries, future work will develop therapies to promote neuromusculoskeletal health and rejuvenation.

## 4 Materials and Methods

### 4.1 MDSPC Isolation

MDSPCs were isolated from skeletal muscle of 21‐day‐old (young) or 2‐year‐old (old) mice via a modified preplate technique, as previously described (Lavasani et al. 2013). Briefly, mouse skeletal muscle is enzymatically digested with collagenase type‐XI, dispase, and trypsin to generate a single‐cell suspension. Cells were passed through a 70 μm filter and separated based on their rate of adhesion to type‐I‐collagen‐coated flasks. The non‐adherent cells were serially transferred to a fresh flask for a total of six passages to isolate the MDSPC population.

### 4.2 Secretome Analysis

Young (*n* = 3) and old (*n* = 3) MDSPCs were cultured under identical proliferation conditions and seeded at 2 × 10^3^ cells per cm^2^ in collagen type I‐coated T175 flasks. Culture media was collected after approximately 26 h of incubation. As a control, fresh proliferation media incubated under the same conditions without cells was also collected to account for background protein levels. Following collection, all media samples were centrifuged at 900×*g* to pellet any possible contaminating cells. The resulting supernatants were subsequently collected and frozen at −80°C. Media samples were then shipped on dry ice to RayBiotech and analyzed using their commercially available Q640 glass slide mouse antibody array (QAM‐CAA‐640‐1).

Quantitative values from each sample, along with the level of detection (LOD) range for each protein, were used for analysis. Proteins with no values exceeding LOD in any sample were excluded from analysis. For the remaining proteins, values below LOD—including the media‐alone background control—were set to the respective LOD. Proteins for which experimental samples (young or old MDSPCs) did not exceed the values detected in media‐alone controls were excluded. To quantify the protein contribution specifically from MDSPCs, background values from media alone samples were subtracted from those of experimental samples; any resulting negative values were set to zero. Triplicate values from young MDSPCs were then compared to the mean of three NA MDSPC populations using the Benjamini‐Hochberg false discovery rate (FDR) method (Benjamini and Hochberg 1995). Functional annotation analysis of the 11 proteins that showed both statistical significance and > 2‐fold higher expression differences, all elevated in the young MDSPC secretome, was performed using the DAVID online bioinformatics resource (Sherman et al. 2022; Huang et al. 2009). The background set for this analysis included the 281 proteins that were above LOD and exceeded media‐alone levels. The DAVID online bioinformatics resource was used to identify associated Gene Ontology (GO) terms, their enrichment *p*‐values, and related factors.

### 4.3 MDSPC Transplantation

All animal experiments were performed with the approval of the Northwestern University Institutional Animal Care and Use Committee. Female C57BL/6 mice were obtained from the National Institute of Aging (NIA) at 20 months of age. At 22 months of age, mice received systemic injections of either 2.5 × 10^5^ young MDSPCs per gram of body weight suspended in 50 μL of PBS or 50 μL of vehicle. A previously isolated well‐characterized population of young MDSPCs that has been utilized in our prior published therapeutic studies (Thompson, Pichika, Lieber, Budinger, and Lavasani 2021; Thompson et al. 2024; Thompson, Pichika, Lieber, and Lavasani 2021; Lavasani et al. 2012) was used to evaluate the therapeutic response in a cohort of naturally aged mice (*n* = 10). Young MDSPCs were administered intraperitoneally (IP), a route previously shown to provide consistent and reproducible outcomes in stem cell transplantation studies (Wang et al. 2016; Al Shoyaib et al. 2019) and used in our prior work (Thompson, Pichika, Lieber, Budinger, and Lavasani 2021; Thompson et al. 2024; Thompson, Pichika, Lieber, and Lavasani 2021; Lavasani et al. 2012). MDSPCs were transduced with a retroviral vector containing *LacZ* with a nuclear localization sequence (n*LacZ*) to detect engraftment in isolated tissues.

### 4.4 Functional Assessment

Open field testing was used to evaluate voluntary locomotor activity and anxiety‐like behavior (Seibenhener and Wooten 2015; Gould et al. 2009). Mice were tested at 23 months of age as previously described (Petrosino et al. 2016; Hu et al. 2019). On the day of testing, mice were acclimated to the testing room 30 min prior to evaluation. For testing, each mouse was placed in an open‐field‐arena (acrylic box: 44 × 44 × 44 cm) and allowed to explore for 10 min. Between trials, the arena was cleaned with 70% ethanol to avoid lingering olfactory cues. Activity in the open field was recorded using a digital video camera and analyzed with Any‐maze software (Stoelting Inc.). Parameters measured included total distance traveled, mean speed, time spent in the central versus peripheral zones, and the immobility duration (resting time in seconds).

### 4.5 Tissue Processing

Body weights of the mice were recorded immediately prior to euthanasia at 24 months of age. Brain and skeletal muscles (gastrocnemius and quadriceps) were surgically isolated, and GS muscle wet weights were recorded. The right GS muscles were flash‐frozen in liquid nitrogen‐cooled 2‐methylbutane for histopathological analysis and the left was flash‐frozen for phosphorylation proteomic analysis. The right quadriceps muscles were preserved in BIOPS solution for immediate respirometry testing and the left was flash‐frozen for citrate synthase analysis. Brains were separated into hemispheres and flash‐frozen in liquid nitrogen‐cooled 2‐methylbutane for histopathological analysis. All tissues were stored at −80°C until processing. Tissues were cryosectioned with a Leica CM1950 cryostat. GS muscles were sectioned into 10 μm thick transverse sections and brains into 10 μm thick sagittal sections, then mounted onto positively charged microscopy slides.

### 4.6 Immunohistochemistry

Muscle fiber cross‐sectional area was assessed by immunohistochemically (IHC) labeling dystrophin (Abcam, ab15277, 1:300), type I muscle fibers (DSHB, BA‐F8, 1:50), type IIa muscle fibers (DSHB, SC‐71, 1:600), type IIb muscle fibers (DSHB, BF‐F3, 1:100), and type IIx muscle fibers (DSHB, 6H1, 1:50), following established protocols (Vella et al. 2011; Lavasani et al. 2014, 2012; Bloemberg and Quadrilatero 2012). Dystrophin images were acquired using a Leica DM6000 automated upright microscope and muscle fiber type images were acquired using an Olympus Slideview VS200, both using a 20× plan APO objective. CSA was quantified in NIS Elements computational software using standardized thresholding parameters for fiber selection and area measurements to ensure unbiased fiber selection and measurement. To evaluate muscle fiber CSA, an average of 500 muscle fibers was quantified across a minimum of three muscle cross sections per mouse (*n* ≥ 7 per group). Muscle fiber–type distribution in GS muscles was assessed by quantifying over 6000 fibers per mouse across at least three muscle cross sections (*n* ≥ 7 per group) using ImageJ.

To evaluate muscle vascularization, GS cross‐sections were double‐labeled for CD31 (BD Biosciences, 553,370, 1:200), a marker of vascular endothelial cells, and dystrophin, as previously described (Lavasani et al. 2012). Images were captured using the same microscope setup, and the capillaries‐to‐muscle fiber ratio was calculated using MuscleJ software's automated vasculature package to minimize bias (Mayeuf‐Louchart et al. 2018). Brain sections were fixed in 4% paraformaldehyde (PFA), then blocked with 10% donkey serum containing 0.3% Triton X‐100 in PBS for 1 h at room temperature. Primary antibodies against CD‐31 and GFAP (BD Biosciences, 553,370, 1:200; Invitrogen PA1‐10004, 1:500), in 3% donkey serum and 0.3% Triton X‐100 in PBS, were applied overnight at 4°C. Following primary incubation, sections were incubated for 1 h at room temperature with secondary antibodies (donkey anti‐rat Alexa Fluor 594, Invitrogen A21209, 1:500; donkey anti‐chicken Alexa Fluor 488, Invitrogen A21207, 1:500) followed by DAPI (1:1000) staining for 15 min. All slides were cover‐slipped using VECTASHIELD Antifade Mounting Media (Vector Laboratories, H‐1000‐10). Brain images were acquired using an Olympus SLIDEVIEW VS200 slide scanner using a 20× Evident X Line objective (UPLXAPO20X). Images were aligned to Allen Brain Atlas reference sections using the BigWarp plugin in ImageJ (Bogovic et al. 2016). Quantitative image analysis was performed in NIS Elements software (Nikon Instruments Inc., AR 5.11.03) using standardized binary layer and thresholding parameters for all brain sections. The motor cortex was divided into two regions of interest (ROIs), superficial (layers 2/3 and 5) and deep (layers 6a and 6b). ROI areas and binary object areas for CD31 and GFAP were measured and exported for analysis.

### 4.7 Histology

Muscle fibrosis was assessed by double‐staining tissue sections with Sirius Red (collagen) and Fast Green (muscle tissue) using a collagen staining kit (Chondrex, #9046) for five minutes. Mosaic light microscopy images were acquired, and fibrosis was quantified using NIS Elements software. Total muscle area and percentage of collagen‐positive area were calculated using consistent threshold parameters across all samples.

### 4.8 Mitochondrial Respirometry

Mitochondrial function in muscle fibers was assessed using high‐resolution respirometry with an Oroboros O2K (Oroboros Instruments, Innsbruck, Austria), following previously established protocols (Dayanidhi et al. 2021; Thompson et al. 2024). Quadriceps muscles were mechanically separated in BIOPS on ice under a dissecting microscope to obtain 2–3 mg fiber bundles for permeabilization and further analysis.

### 4.9 Citrate Synthase Rate Assay

Pulverized tissue was homogenized in Zheng buffer and centrifuged at 600×*g* for 10 min at 4°C, as previously described (Thompson et al. 2024; Dayanidhi et al. 2021). Protein concentration in the supernatant was measured using the Pierce bicinchoninic acid (BCA) protein assay kit, following the manufacturer's instructions. Enzymatic activity was assessed using 10 μg of protein per well by monitoring absorbance at 412 nm over 3 min, with an extinction coefficient of 13.6 mmol/L/cm.

### 4.10 Angiogenesis Phosphorylation Analysis

Phosphorylation of key protein sites involved in angiogenesis was analyzed in GS muscle tissue. Whole GS muscles (> 100 mg) were shipped to Full Moon Biosystems where samples were processed and applied to their VEGF pathway phospho‐antibody glass slide array (cat# PVE185). Phosphorylation levels were normalized according to the manufacturer's instructions using the median signal intensity of each array. Comparisons were made between phosphorylation sites probed by the same antibody at the same array location.

### 4.11 Statistical Analysis

Statistical analyses were performed using SigmaPlot (Jandel Scientific v14.0) and GraphPad Prism (v9) software packages. Depending on the distribution and variance characteristics of the data, appropriate statistical tests were applied, including the one‐tailed unpaired Welch's *t*‐test (for unequal variances), one‐tailed Student's *t*‐test, Mann–Whitney rank‐sum test, or the nonparametric two‐tailed Kolmogorov–Smirnov test. Violin plots illustrate the median and upper and lower quartiles, while bar graphs, dot plots, and frequency distributions are presented as the mean ± SEM. Statistical significance was defined as *p ≤ 0.05, **p ≤ 0.01, ***p ≤ 0.001 and ^§^ *p* ≤ 0.0001. Values with *p* ≤ 0.07 were considered to indicate a strong trend and are marked with a plus symbol (+).

## Author Contributions

Seth D. Thompson and Mitra Lavasani conceptualized the study, performed in vivo cell transplantation experiments, acquired and analyzed data, and contributed to data interpretation. Chelsea L. Rugel conducted brain tissue sectioning, imaging, histological analysis, and drafting the brain‐related section of the manuscript. Jodi L. Curtin and Maddlyn R. Haller carried out muscle tissue sectioning, staining, imaging, histological analysis, and supplementary figure preparation. Sudarshan Dayanidhi performed and oversaw data collection, statistical analysis, data interpretation, and manuscript editing for respirometry experiments. Seth D. Thompson and Mitra Lavasani supervised the project, prepared figures, and wrote the manuscript. All authors reviewed and approved the final version of the manuscript.

## Funding

This work was supported by the Lisa Dean Moseley Foundation, Julius N. Frankel Foundation, Shirley Ryan AbilityLab Innovative Catalyst Grant, and NIA‐R01 AG073223 to M.L.

## Conflicts of Interest

The authors declare no conflicts of interest.

## Supporting information


**Data S1:** acel70408‐sup‐0001‐FigureS1‐S2.pdf.

## Data Availability

Data will be made available on request.
