# Supplementary material for: Secretome Profiling of Young Multipotent Stem Cells Reveals Angiogenic and Immunomodulatory Mechanisms Supporting Aged Neuromuscular Health
Source: Aging Cell. 2026 Feb 12;25(2):e70408. doi: 10.1111/acel.70408 (PMC12895213; doi:10.1111/acel.70408)
Supplement: Supplementary file 1 — Data S1: acel70408‐sup‐0001‐FigureS1‐S2.pdf. [file ACEL-25-e70408-s001.pdf]

Secretome Profiling of Young Multipotent Stem Cells Reveals Angiogenic and Immunomodulatory Mechanisms Supporting Aged Neuromuscular Health

Seth D. Thompson*, Chelsea L. Rugel, Maddlyn R. Haller, Jodi L. Curtin, Sudarshan Dayanidhi, Mitra Lavasani*

***Address correspondence to:**
Seth Thompson (seththompson2018@u.northwestern.edu) or
Mitra Lavasani (mlavasani@sralab.org, mitra.lavasani@northwestern.edu)

Supplementary Figures

Supplemental Figure 1: Body and muscle weights of 24-month-old naturally aged mice treated with young MDSPCs or PBS. (**A**) Average body weights of mice systemically injected with young MDSPCs (NA-CI; *n* = 9) or PBS (NA-PBS; *n* = 7). (**B**) Average gastrocnemius (GS) wet weights normalized to body weight. (**C**) Quantification of mitochondrial respiration rates in quadriceps muscle tissue. (**D**) Representative image of GS muscles labeled for type I (blue), type IIa (green), and type IIb (red) muscle fibers at two months transplantation. (**E**) Representative image of a consecutive GS muscle section from Fig. S1D, labeled for type I (blue), type IIa (green), and type IIx (red) muscle fibers, validating the identification of unlabeled fibers stained in the method of Fig. S1D as type IIx fibers. Data are presented as mean ± SEM. Statistical significance was determined using one-tailed unpaired Student's *t*-test. Scale bars are 100 µm (D and E).

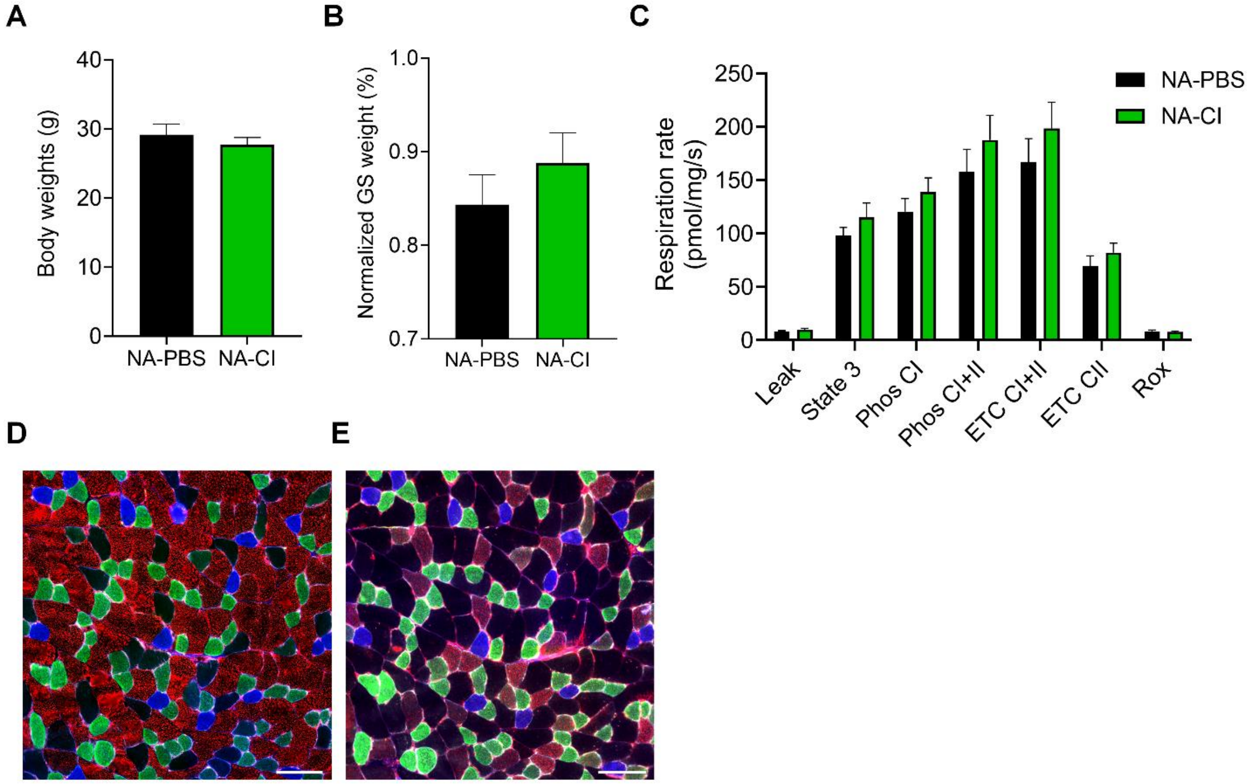

Supplemental Figure 2: Determination of donor cell engraftment following intraperitoneal transplantation of young MDSPCs. Top left images represent positive control gastrocnemius (GS) muscles stained with X-gal, to detect donor *LacZ*+ young MDSCPs (blue) three days post-intramuscular transplantation with eosin counterstaining. Remaining images representant GS, heart, lung, brain, liver, bladder, kidney, pancreas, spleen, thymus, and lymph nodes from naturally aged mice systemically injected with young MDSPCs (NA-CI) stained with X-gal and counterstained with eosin. Scale bars; 1 mm (inset: 200 μm).

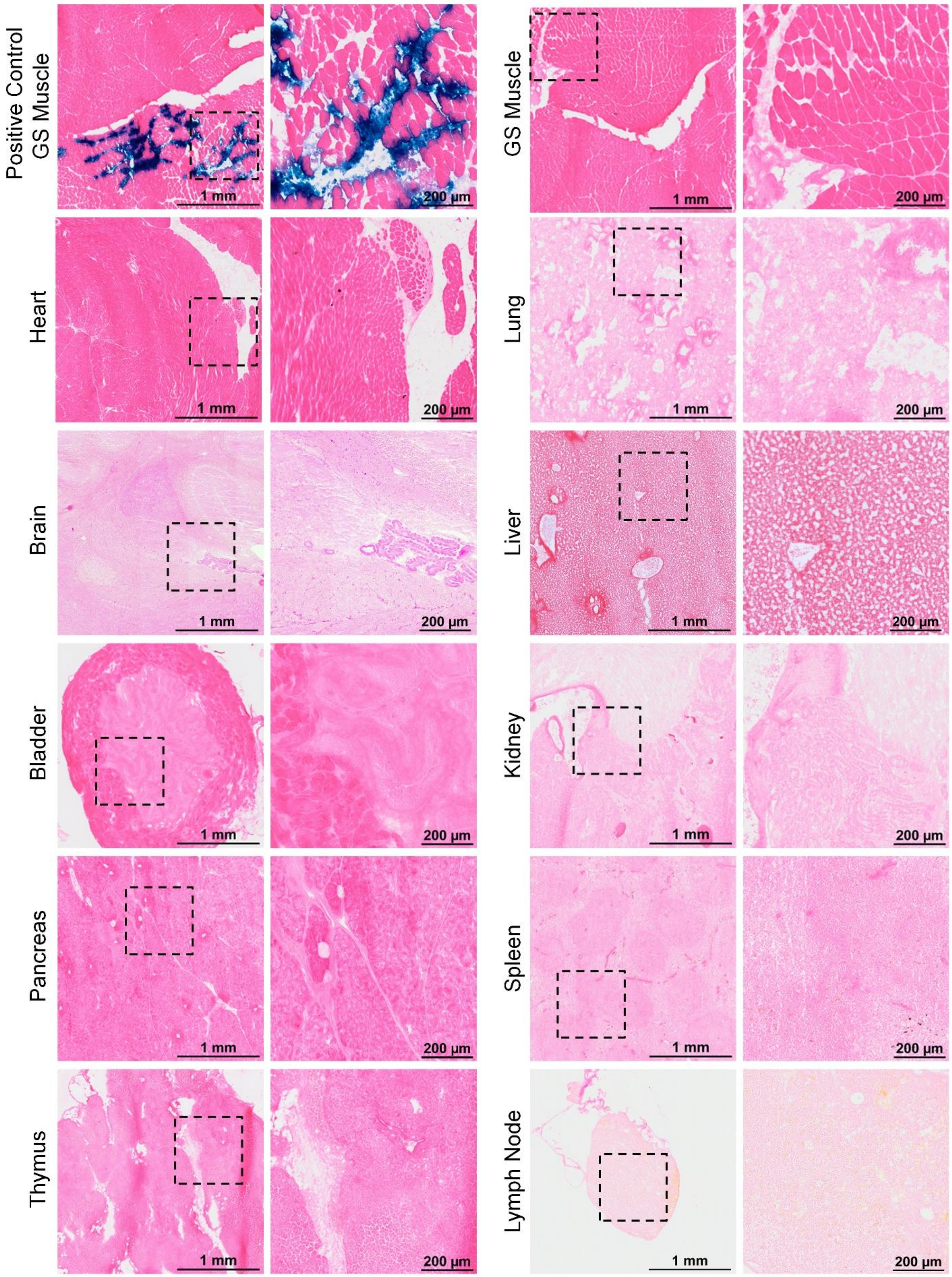
Positive Control
GS Muscle
1 mm
200 µm
GS Muscle
1 mm
200 µm
Heart
1 mm
200 µm
Lung
1 mm
200 µm
Brain
1 mm
200 µm
Liver
1 mm
200 µm
Bladder
1 mm
200 µm
Kidney
1 mm
200 µm
Pancreas
1 mm
200 µm
Spleen
1 mm
200 µm
Thymus
1 mm
200 µm
Lymph Node
1 mm
200 µm
